# Crossing borders, shaping politics: A study on the political interest of Chinese international student returnees of Eastern China

**DOI:** 10.1371/journal.pone.0333198

**Published:** 2025-09-26

**Authors:** Ruining Jin, Boya Wu, Tam-Tri Le

**Affiliations:** 1 Institute of Higher Education, Beijing University of Technology, Beijing, China; 2 Capital Engineering Education Development Research Base, Beijing, China; 3 Civil, Commercial and Economic Law School, China University of Political Science and Law, Beijing, China; 4 Independent Researcher, Ho Chi Minh City, Vietnam; National Cheng Kung University, TAIWAN

## Abstract

The political interest of highly educated returnees represents a significant force in shaping the political landscape and bridging cultural and political divides. Conducting Bayesian analysis aided by Markov Chain Monte Carlo (MCMC) algorithms on a sample of 1014 returnee participants, this research probed the influence of sociodemographic and socio-psychological factors on political interest of the Chinese international student returnee population. The findings reveal that education attainment, age, and individualistic values negatively correlate with political interest, while time spent abroad and the intention to emigrate positively affect returnees’ interest in politics.

## 1. Introduction

### 1.1. Political interest and its determinants

Politics can be defined as a combination of conflict resolution, the art of government, the conduct and management of public affairs, and more [1,2]. Political interest, on the one hand, refers to one’s motivational factor that leads to political engagement [3,4]. On the other hand, because one who has political interest does not necessarily take action accordingly [5], political interest can also be understood as a feeling stemming from a sense of citizenship duty. Combining these definitions, political interest in the current study refers to an individual’s psychological state of curiosity, attentiveness, and engagement toward political affairs, issues, and processes.

Interest in politics can be genetically determined, as prior genopolitical studies substantiated the influence of genes on individuals’ political attitudes and behavior, proposing that genetic differences can significantly explain variations in political behavior beyond social factors [6–8]. Sociodemographic factors also play a crucial role in shaping individuals’ interest in politics, including education [9–12], socioeconomic status [13], age [14], and the influence of social networks [15,16].

However, it has been noticed that there has been a decreased political interest among the younger generation on traditional political issues [12,17–20]. Some argue that current model of political engagement (such as voter turnout) did not resonate well with the younger generation’s preferred electronic participation, leading to the perceived low interest and participation in politics [14,18]. Others concluded that younger generation’s perceived low political interest stems from the disparity between their preferred sociopolitical identity and the political reality [12,20].

### 1.2. COVID-19 and its impact on political socialization in China and the West

Given that younger generations are more likely to be ideological liberalists [21], one possible reason for the abovementioned disparity that caused their disinterest in politics might be the conservative political trend associated with COVID-19 pandemic. Specifically, during the COVID-19, nationalist sentiments and inward-looking policies have been observed as a global phenomenon [22]. In this scenario, right-wing ideologies surged, providing fertile ground for populist movements advocating restrictive immigration policies and prioritizing native populations [23]. Budgetary reductions in education funding following the economic downturn caused by COVID-19 endangered the protection of underrepresented groups [24]. In this context, governments around the globe are trying to capitalize on and further institutionalize nationalist sentiments, integrating them into public policy and political socialization processes.

For example, in the West, during and after COVID-19, traditional Western political socialization (limiting government power, realizing institutional separations, and promoting popular participation [25], as well as instilling individualized values such as freedom, civic engagement, and rule of law [26,27]) gave way to the promotion of patriotic education [28], centralization of power within the executive branch [29], and reductions in funding for college programs that uphold Diversity, Equity, and Inclusion (DEI) as well as Critical Race Theory (CRT) initiatives [30–33].

In China, political socialization through education mainly centers on the unity and cohesion of the country and stresses the function of “etiquette” in education, uniting political socialization with political recruitment [34]. Through the integration of political socialization in various levels of the education system, the country promotes the socialization of socialist politics and values with Chinese characteristics to enhance the system’s spiritual realm and moral legitimacy [35,36]. At a macro level, the core idea of political socialization is to promote collectivistic values such ingroup cohesion, mutual understanding, and peaceful coexistence [37]. During and after the COVID-19 period, there was a notable surge in nationalist sentiments among Chinese youth, largely attributed to reinforced patriotic education in college for faculty and students [38–40]. These patriotic education and political socialization culminated in positive evaluations of China’s pandemic response and negative perceptions of other nations’ handling of the crisis [41,42]. While the patriotic education might function to enhance national pride and unity, it also has side effects: xenophobic sentiments on Chinese cyberspace prevailed, leading to strong cybernationalism in the cyberspace [43], and hate crimes against foreigners in the post-COVID-19 era [44–46].

### 1.3. The double political socialization on Chinese international student returnees

Growing up in China and going through education in both domestic and overseas institutions, Chinese international students would go through a double political socialization, given by domestic and foreign education institutions as well as societal influences. As a result, such a double political socialization might lead to interesting yet nuanced outcomes of their political interest. On the one hand, patriotic education and the address for a stronger ingroup preference in both the home and host societies might form a Social Dominance Orientation (a psychological term illustrating a preference for maintaining hierarchical structures in domestic and global settings) [47,48], increasing their political interest in future political events. For example, it has been documented that first-generation Chinese immigrants have voiced in their fervent, yet seemingly paradoxical support for Donald J Trump, who held anti-immigration, anti-China, and Social Dominance Oriented views during all three presidential bids to the White House [49,50].

On the other hand, double political socialization might also offer Chinese international students an increased level of sensitivity and understanding regarding different cultural communication styles and societal norms. These acquired knowledge and experience would enhance their cross-cultural awareness and cultural proficiency [51]. Moreover, during collaborative learning activities of political socialization, they might also acquire enhanced analytical skills to help form a balanced judgment based on newly gained group membership. This would cultivate their political activism, problem-solving skills, and decision making as future leaders in their later careers [52–54]. For example, many Chinese international students have been observed to display cosmopolitan values, actively participating in demonstrations and rallies to show empathy towards the weak and the minority groups, highlighting their political dissent towards the host nation’s stance on certain regional conflicts [55,56].

Alternatively, however, exposure to different political socializations can also lead to identity conflicts and cognitive dissonance [57,58]. Prior studies have indicated that group disagreements might lead to cognitive dissonance, which would impair the mental health conditions of international students [59]. In particular, because COVID-19 led to massive discrimination in the host nation towards Chinese international students, as well as pervasive Sinophobia, Chinese international students’ political interest could also decrease as a response to these social conditions. Prior studies have suggested that because of discriminations from the host nation, Chinese international student tend to separate themselves from the host nation’s education institution and society, seeking support from other co-nationals, and withdrawing from host nation’s social media to avoid discrimination [60–62].

Given these complexities, it is imperative to discuss the significance of political interest among Chinese returnees, as their engagement holds substantial implications not only for their own integration but also for broader societal and global contexts.

### 1.4. The importance of political interest among returnees

Political interest among returnees is crucial because it serves as a foundation for active and meaningful participation in public affairs, influencing their ability to advocate for themselves and others in society. From the perspective of Theory of Planned Behavior [63], positive attitudes towards certain practices might increase the chance of actually conducting such practices. In this line of thinking, interest in politics might translate into greater political engagement, which would be vital to both the returnee population and the broader society.

In fact, the international student returnee population does need to maintain a high political interest so they can always make their voice heard in both host and home nation environments to safeguard their rights and those who are underrepresented based on prior cases. For example, a Chinese student’s Student Visa was revoked for her participation in political demonstrations against the host nation’s attitude towards certain regional conflicts [56]. International students from other nations, such as the case of Dana Abuqamar and Mahmoud Khalil, who expressed their dissent on the host nation’s foreign policies regarding certain regional conflicts, also suffered from persecutions from host nation authorities and right-wing politicians [64,65]. Moreover, Chinese international student returnees, during their abroad and return experience, also endured discrimination and stigmatization from the home country [66,67]. In 2012, two Chinese international students were killed while overseas. Due to their affluent backgrounds, domestic media attention disproportionately emphasized the luxury car they were driving, thereby shifting public criticism from the violence itself to perceptions of their privileged lifestyle [68]. Furthermore, ideological tensions between China and Western countries have also contributed to discrimination against Chinese international students perceived to align with Western values. For instance, a female returnee faced severe public backlash and stigmatization as a “giant infant” after demanding human rights during COVID-19 hotel quarantine [58,69]. Similarly, in 2017, another Chinese international student experienced intense online harassment, stigmatization, and doxing from Chinese netizens after praising the superior air quality in the United States that that of China during a commencement speech [70,71]. In April 2025, Chinese famous female entrepreneur Dong Mingzhu explicitly declared that her company would not hire any returnees because “there might be spies among them” [72], further exemplified the phenomenon of domestic discrimination faced by returnees in contemporary China.

To address previously mentioned challenges, greater political engagement might help voice their concerns in public affairs and policy implementation. This would help reduce bias and promote smoother integration in a target society. From a societal perspective (China), returnees’ greater political engagement might help China retain more talent and avoid the crisis of brain drain in the post-COVID-19 era [73,74]. Furthermore, from a global perspective, a larger talent pool composed of these “cultural brokers” in any given society might help play pivotal roles in domestic and foreign affairs, such as reducing misunderstandings [75], easing bilateral tensions [76], and fostering increased collaboration across diverse cultures [77,78]. Lastly, from a humanitarian perspective, greater political engagement among returnees can help promote the United Nation’s Sustainable Development Agenda and facilitate “global development and of ‘win-win’ cooperation which can bring huge gains to all countries and all parts of the world” [79].

Based on the discussion above, the current study aims to find the factors influencing Chinese international student returnees’ political interest, so more information about the demographic and possible underlying psychological patterns affecting political interest among this population can be discerned.

## 2. Methodology

### 2.1. Materials and variables

The study sampled 1014 Chinese international student returnees of Eastern China from five WeChat returnee public groups (Beijing, Shanghai, Suzhou, Shenzhen, and Guangzhou). In the context of this study, “Chinese international student returnees” specifically refers to individuals who were born and raised in Mainland China, pursued their overseas education abroad in the West, and subsequently returned to Mainland China. The survey collection was between Oct 8, 2023, and Jan 30, 2024. The inclusion criteria for this survey are: 1) participants must be born and raised in China and had overseas education experience; 2) returned China after their overseas experience; 3) had stayed in China for more than 1 year after their reentry; and 4) have not taken part in the same survey in other WeChat public groups. Given that during and after the COVID-19, there were large-scale emigrations of Chinese highly skilled laborers [73,74], therefore those who were qualified based on abovementioned criteria but had already left China for Western countries would also count. The survey questions were programed in WeChat’s MiniApp *SurveyStar*. Before the survey, researchers shared the purpose of the study, inclusion criteria, along with the informed consent to the city-based WeChat returnee public groups. After several rounds of screenings, the researcher received 1014 valid responses. Nevertheless, the reliance on WeChat groups introduces potential selection biases. Specifically, individuals active in such groups may possess distinct socioeconomic characteristics and transnational identity clusters, as one prior study indicated that the use of social media platforms (popular in China vs. popular in the West) were associated with their cultural belongings and integration levels in the host nation [80]. Such biases could affect sample representativeness, thereby limiting the generalizability of study findings. This limitation will be further elaborated upon in the study’s limitation section.

The studies involving human participants were reviewed and approved by the Institutional Review Board at China University of Political Science and Law. The survey was anonymous and did not contain any information that would compromise the confidentiality of participants’ identity, and written informed consent was received from all participants before their survey participation. Table 1 presents the variables from the dataset that are used in this study.

**Table 1:** Variable description.

Variable name	Meaning	Value
*Politics*	Participants’ self-reported psychological level of interest in politics	1. None
		2. A little
		3. Moderately
		4. Strongly
		5. Extremely
*Emigrate*	The degree of the participant’s intention to emigrate	1. Definitely stay
		2. Likely stay
		3. Unsure
		4. Likely migrate
		5. Definitely migrate
*TimeAbroad*	Total time the participant has spent in a foreign country	1. < 1 years
		2. 1–2 years
		3. 2–5 years
		4. 5–10 years
		5. > 10 years
*Education*	The participant’s highest educational attainment	1. Elementary or lower
		2. Secondary School
		3. High School
		4. Undergraduate
		5. Postgraduate or higher
*Individualist*	To what degree one views oneself as an individualist	1. None
		2. A little
		3. Moderately
		4. Strongly
		5. Extremely
*AgeGroup*	The participant’s age group	1. < 18
		2. 18-30
		3. 31-40
		4. 41-50
		5. > 50

*Note: All participants in this sample are over 18 years old.

Table 1 has highlightes the definitions of *Politic, Emigrate,* and *Education.* All variables are categorical variables. Variable *Politics* refers to participants’ self-reported psychological level of interest in politics, defined specifically as their internal state of curiosity, attentiveness, and cognitive engagement with political affairs, issues, or processes. Variable *Individualist* denotes to what degree one views oneself as an individualist as opposed to a collectivist. Variable *TimeAbroad* measures participants’ total time spent in a foreign country, where “1” means “less than one year”, “2” means “1-2 years”, “3” means “2-5 years”, “4” means “5-10 years”, and “5” means “more than 10 years”. Lastly, *AgeGroup* refers to participants age group, where “1” means participants is below 18, “2” means participants is in the 18–30, “3” means the participants age in their 31–40, “4” means participants are in the 41–50, and “5” means older than 50.

The participants primarily aged between 18 and 30 years (61.74%), with a smaller proportion in the 31–40 (28.80%) and 41–50 (9.47%) age groups. Among all participants, a notable majority expressed a strong interest in politics, with 31.07% showing strong interest and 38.95% extremely strong interest, while only 15.98% reported no interest. Regarding overseas stays, the distribution was fairly even across different durations, with the longest stays (over 10 years) slightly leading at 27.22%. Educationally, nearly half of the respondents completed undergraduate degrees (49.90%), and a significant portion attained postgraduate degrees (24.65%). On the question of emigration intent, opinions varied, with 38.46% undecided and a substantial number leaning towards emigration (27.02% definitely and 25.15% likely). When asked about individualistic values, a moderate stance was most common (38.46%), followed by extreme (27.02%) and strong (25.15%) identification with individualism.

### 2.2. Analysis procedure

In this study, Bayesian analysis aided by Markov Chain Monte Carlo (MCMC) algorithms was used. We use the following formula of the analytical model:

$\mu_{i}=\beta_{\text{0}}+\beta_{Emigrate}*{Emigrate}_{i}+\beta_{TimeAbroad}*{TimeAbroad}_{i}+\beta_{AgeGroup}*{AgeGroup}_{i}+\;\beta_{Educaation}*{Education}_{i}+\beta_{Individualist}*{Individualist}_{i}$

$\mu_{i}$ represents the interest level in politics of returnee i with posterior estimations in the form of normal distribution. Returnee i’s age group is ${AgeGroup}_{i}$. Returnee i’s educational attainment is ${Education}_{i}$. Returnee i’s emigration intent is ${Emigrate}_{i}$. Returnee i’s agreement to individualism is ${Individualist}_{i}$. Returnee i’s time spent abroad is ${TimeAbroad}_{i}$. The model has an intercept $\beta_{\text{0}}$ and coefficients $\beta_{Emigrate}$, $\beta_{AgeGroup}$, $\beta_{Education}$, $\beta_{TimeAbroad}$, and $\beta_{Individualist}$.

For statistical analysis, we used Bayesian analysis with aided Markov Chain Monte Carlo (MCMC) algorithms. The technical reasoning, analysis procedure, and result presentation follow the protocol of MCMC-aided Bayesian analytics for social sciences and psychological research [81,82]. Bayesian analysis aided with MCMC allows us to simulate many possible outcomes from our sample, which enhances the accuracy of our conclusions when comparing to traditional methods. However, the reliability of the simulated data (or “chains”) need to be tested, so we check their performance using three key indicators: 1. The technique of Pareto-Smoothed Importance Sampling Leave One-Out (PSIS-LOO) diagnostics was used [83,84]. This approach examines how well our model predicts the data based on the value of *k* value for each data point. If all *k* values are under 0.5, then it indicates that our model fits well; if the *k* values are above 0.7, then this would mean potential problems. 2. In addition, we also use *Effective Sample Size (n_eff)*. This can be considered as the number of independent data points our simulation represents. If *n_eff* values are above 1000, it then suggests that our estimates are based on enough ‘good’ samples [85]. 3. Thirdly, *Gelman-Rubin Shrink Factor (Rhat)* is another key indicator. This statistic tells us if our different simulation runs (chains) have settled to similar values. In this regard, an *Rhat* value of 1 (*Rhat* = 1) means the chains have converged and our results are stable [86]. Overall, if a model’s *k* value is smaller than 0.5, *n_eff* is larger than 1000, and *Rhat* equals 1, then the model is deemed reliable. Robustness testing [87] in the frequentist sense is not suitable for the current study, and our modeling approach follows the standard procedures outlined in Bayesian Mindsponge Framework guidelines [82], which has been widely used in social science research among various fields [88–91]. We use the bayesvl package [92] in R to conduct this study. Markov chain convergence can also be assessed through visual approaches such as trace plots, Gelman-Rubin-Brooks plots, and autocorrelation plots. The MCMC setup is comprised of 5000 total iterations, with 2000 warm-up iterations and 4 chains.

## 3. Results

**Fig 1:**
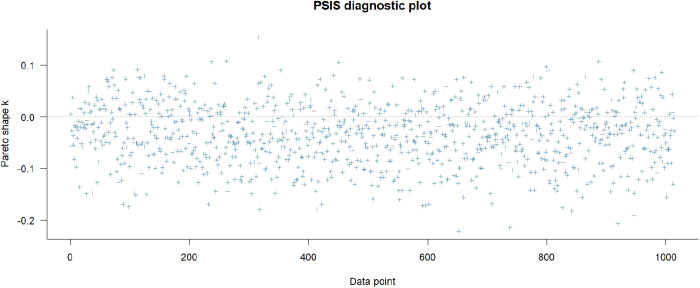
PSIS diagnostic plot.

Fig 1 below is the PSIS diagnosis result. It can be observed that all *k* values are lower than the threshold of 0.5, which could be seen as a sign of goodness-of-fit.

**Fig 2:**
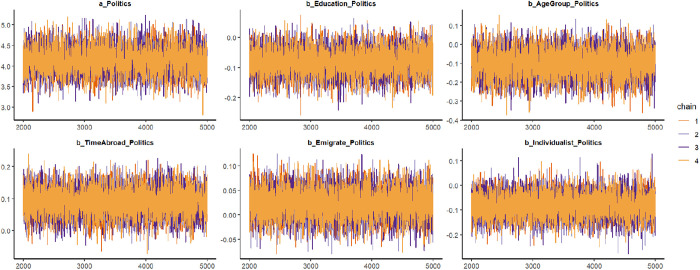
Trace plots.

The findings are listed in Table 2. The statistical analysis shows a good convergence of the model’s Markov chains, given that the effective sample size (*n_eff*) exceeds 1000 and the Gelman-Rubin shrink factor (*Rhat*) is 1, which demonstrates acceptable reliability of the posterior coefficients. In Fig 2’s trace plots, colored lines represent the Markov chains. Line fluctuations can be observed around a central equilibrium after the warmup period, so it is a good indicator of well-mixing and stationary qualities.

**Table 2:** Simulated posteriors.

Parameters	Mean (M)	Standard deviation (S)	*n_eff*	*Rhat*
Constant	4.14	0.31	6006	1
*Education*	−0.08	0.04	8786	1
*AgeGroup*	−0.10	0.07	8746	1
*TimeAbroad*	0.09	0.05	9309	1
*Emigrate*	0.02	0.03	10944	1
*Individualist*	−0.08	0.05	8584	1

**Fig 3:**
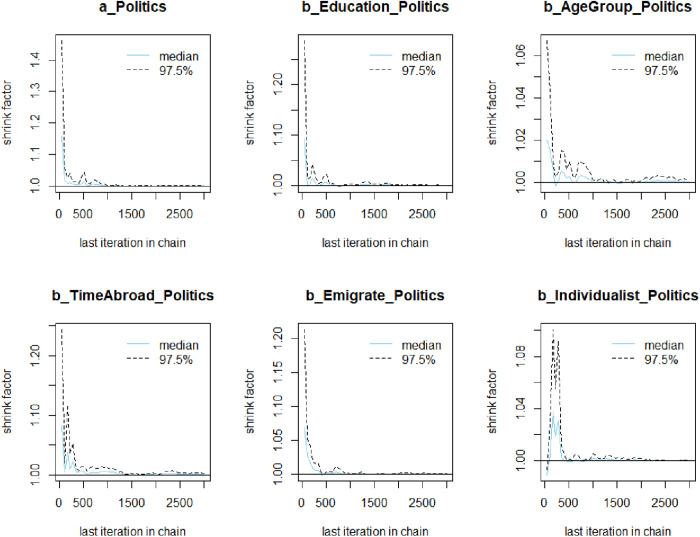
Gelman-Rubin-Brooks plots.

**Fig 4:**
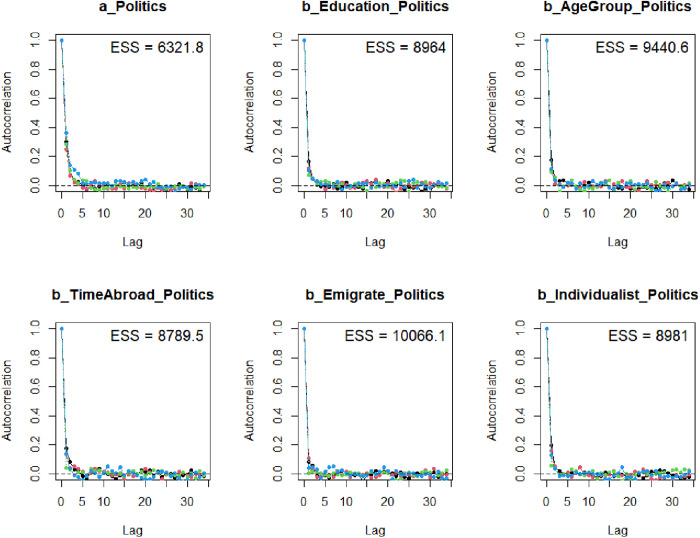
Autocorrelation plots.

The Gelman-Rubin-Brooks plots (Fig 3) indicate that *Rhat* values drops quickly to 1 in the warm-up period. The autocorrelation plots (Fig 4) also suggest a quick elimination of problematic autocorrelation among simulated data points within the MCMC processes.

**Fig 5:**
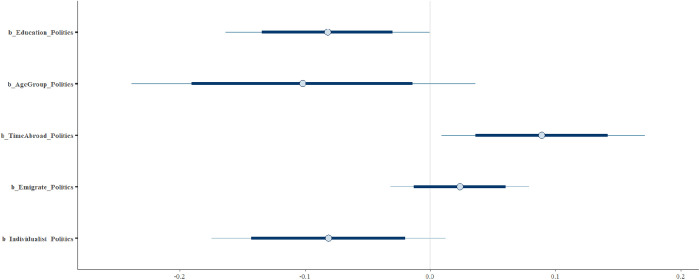
Posterior distributions.

According to the analysis results, *AgeGroup, Education*, and *Individualist* have a clear negative association with returnees’ level of political interest *Politics* ($M_{AgeGroup}\;=\;-\text{0}.\text{10}$ and $S_{AgeGroup}\;=\;\text{0}.\text{07}$, $M_{Education}=-\text{0}.\text{0}\text{8}$, $S_{Education}=\;\text{0}.\text{04}$, $M_{Individualist}\;=\;-\text{0}.\text{08}$ and $S_{Individualist}\;=\;\text{0}.\text{05}$). On the other hand, Individual’s emigration intention *Emigrate* has a positive association with their level of interest in politics (moderately reliable) ($M_{Emigrate}\;=\;\text{0}.\text{02}\;$ and $S_{Emigrate}=\;\text{0}.\text{03}$); *TimeAbroad* has a clear positive impact on individuals’ interest in politics ($M_{TimeAbroad}=\text{0}.\text{0}\text{9}\;$and $S_{TimeAbroad}=\;\text{0}.\text{05}$). In Fig 5, it can be observed that the posterior distributions of *AgeGroup, Education*, and *Individualist* lie almost entirely on the negative side. On the other hand, *TimeAbroad* lies entirely on the positive side. Moreover, the posterior distributions of *Emigrate* also mainly lies on the positive side.

## 4. Discussion

The multi-regression analysis results show the relationships between political interest and some sociodemographic/socio-psychological factors. We found that education attainment, age group, and individualistic value prioritization have a clear negative association with participants’ political interest. Participants’ overseas-staying duration has a clear positive association with political interest. There is also a positive association between political interest and one’s emigration intention (low-moderate reliability).

### 4.1. Education

The current study is in alignment with prior study findings that the education attainment increase does not automatically lead to increased political interest [9,11]. One possible explanation is that a majority of Chinese students are majoring in STEM and business subjects during overseas studies, especially at the graduate level [93,94]. Host nations’ immigration policies, such as the United States’ Optional Practical Training (OPT) program, grant STEM graduates a 24-month extension for practical training [95], and the H-1B work visa program favors individuals in specialized occupations, including STEM fields [96,97]. These policies increase the likelihood of STEM students remaining abroad, encouraging more international STEM students to pursue study overseas in the West. Conversely, students in humanities and social sciences face more challenges in securing opportunities to stay abroad, partly due to limited extensions like the STEM OPT. Also, because of the consumerization of higher education of business schools in the West [98,99], many business programs usually prioritize attracting international students due to their financial contributions through higher tuition fees. This structural bias may systematically exclude students who are more likely to be interested in politics, as they often belong to fields more closely aligned with political engagement. As a result, the higher their education attainment level, the more likely Chinese international STEM and business students might become more specialized and narrowly focused on their professional knowledge/skills in their daily activities (especially professional jobs). In this regard, their professional-oriented focus (which is less relevant to politics in general) might lead to less time and energy for interest outside their immediate academic or professional interests, including politics.

### 4.2. Age group

The current study’s findings on age contradict with prior research in Western countries, where political interest grows as individuals age [100,101]. In this case, Confucian’s role ethics might help explain such a disparity. In Confucian role ethics, humans’ moral behavior in various contexts is shaped by the unique roles they assume in those settings. Individuals adopt distinct roles that are influenced by their connections with other people, so various interpersonal connections and societal positions influence one’s decision-making [102]. Based on this philosophy, returnees might develop more roles and concerns as they age—in addition to being the children of their old parents, older returnees are more likely to become parents, spouses, primary caregivers, and income makers, taking more familial responsibilities and challenges. Thus, their interest and attention might shift toward more concrete and pragmatic issues, such as income and health worries. However, as role conflict theory suggests, individuals occupy various roles within social structures, each with distinct sets of norms and expectations. When the demands of these roles conflict, individuals may prioritize roles based on their significance, personal values, or societal expectations, leading to the adjustment or abandonment of roles that are less critical to their identity or well-being [103]. In this case, influenced by Confucian social norms and group harmony, those Chinese returnees who prioritized their familial role over social role might have to be occupied by family concerns, thus becoming less interested in politics.

### 4.3. Time Abroad

The findings indicate a positive association between the duration of overseas stay and the political interest of returnees. One possible explanation is that, Chinese domestic media and social media tend to view Western democracy through a negative light [104–106], therefore, Chinese international students’ initial interest in politics could be shaped through these pre-departure political socialization. However, as they experience prolonged exposure to politics abroad, some of them might develop a clearer and more balanced understanding of Western democratic processes, thereby stimulating greater curiosity and interest in politics. Also, because of the escalated China-West relationships in recent years, Western media has largely demonized China in their media coverage [107,108]. While in some cases, “China-bashing” might cause cognitive dissonance, social media withdrawal, reliance on co-nationals, and decrease of interest in politics [60–62], extended exposure by to Western media propaganda by prolonged overseas staying might also heighten their awareness of international politics. This awareness would potentially spark a more critical and engaged political consciousness. For example, earlier studies also found that Chinese international students, after overseas studying, developed a stronger interest in politics that also viewed Western democracy through a critical lens [109,110].

### 4.4. Individualist

In general, individualistic value prioritization is marked by their association with greater concerns about the well-being [111], and thus they are probably less likely to pay attention to politics, including political figures, public policies, collective campaigns, etc. However, in the Chinese sociopolitical context where individualism and economic affluence are intertwined [112,113], resources such as time, social capital, money, and attention might be allocated more toward personal financial success. Such a resource allocation might be driven by Chinese pragmatism ideology and value prioritization since the open-up policy [114]. Also, although the trend of individualization and economic prosperity was widely noticed in the late 90s and early 2000s [112], the current Chinese administration is attempting to restore traditional values, including collectivism and nationalism [35,115,116]. Such value prioritizations might be inconsistent with individualistic-oriented returnees, which would further lower their political interest. One study on the Chinese post-1980s generation’s political attitude offered similar findings, suggesting the Chinese younger generation’s less interest in collectivized/nationalist value prioritization, and a generally low interest in politics [20].

### 4.5. Emigrate

Chinese returnees who have the intention to emigrate could be defined as transmigrants who might maintain networks and activities encompassing both their host and home countries. Therefore, during their overseas they probably develop a global mindset as their lives cut across national boundaries, connecting two societies into a single social field [117]. So, they might form a political interest in politics in China as well as other global powers, observing the global political landscape to better guide their venture and professional careers. Because of the transnationalism and transmigrants mentioned above, they may also form transnational identities between the host country (ies) and the home country. Because of such transnational identities, they might have sociocultural/sociopolitical identity and value conflicts, which might reinforce their desire to change living environments [74,118]. In this scenario, such identity conflicts can make them pay more attention to national and foreign politics, which can affect their current and future living environments.

### 4.6. Implications

Based on the research findings, there are several implications. First of all, it is suggested that educational institutions, both domestic and overseas, may consider offering interdisciplinary courses that combine specialized technical content with social science subjects to foster a broader understanding and interest in political and social issues among undergraduate and graduate students. This would direct students to critically apply their knowledge and skills learned on campus to political discourses, increasing their interest in politics. Secondly, given that returnees age is negatively associated with their political interest, policymakers should create various channels that can engage older returnees and increase their political interest. It is suggested that policymakers might consider offering flexible engagement opportunities that accommodate their busy schedules and familial obligations. Prior studies have indicated that online participation such as social media political participation, can function effectively to engage users [119,120]. Thirdly, considering the positive impact of time spent abroad on political interest, the value of international experiences should be underscored in the hope of broadening individuals’ worldviews and fostering a global perspective. Consequently, governments should offer policy incentives to support young individuals’ participation in international exchange programs, as such experiences can be a strategic approach to enhance political interest and engagement among the youth and returnees alike. Fourthly, the negative association between individualistic values and political interest underscores the need for dialogue and educational efforts that bridge these value orientations, promoting an inclusive society that balances individual aspirations and collective responsibilities. Lastly, due to the positive association between emigration intent and political interest, there is a complex relationship between transnational identities, sociopolitical engagement, and life choices. Therefore, it is suggested that societies should address the unique needs and perspectives of transmigrants, ensuring that their potential contributions to both their home and host societies are recognized and facilitated.

### 4.7. Limitations

The study is not without limitations. The reliance on WeChat groups for participant recruitment, which may not represent the broader population of returnees. The use of self-reported data could introduce response biases, and the cross-sectional design limits the ability to infer causality or track changes over time. Additionally, the study’s focus on a specific demographic and its quantitative methodology may overlook broader socio-political factors and the nuanced experiences of returnees. Furthremore, this study focuses explicitly on subjective self-reported political interest due to the politically sensitive context of Mainland China. While objective measures of political participation—such as active involvement in political activities, public discussions, or voting behaviors—could provide complementary insights, these indicators might introduce bias or response inaccuracies given participants’ potential hesitancy to report such behaviors. Thus, our narrower operationalization of political interest ensures greater reliability and honesty in responses, despite the inherent limitation of not capturing actual participatory behavior. Finally, we note that our operationalization of regional background is based on self-reported WeChat group membership, which may not accurately reflect respondents’ actual place of residence or political socialization. As group membership is not strictly tied to the current domicile, this introduces potential misclassification and precludes the reliable inclusion of region as a control variable. Future research could address these gaps by incorporating diverse sampling methods, longitudinal designs, and qualitative analyses, thereby providing a more comprehensive understanding of political engagement among returnees.
